# New-Onset Diabetes After Pancreaticoduodenectomy: 3D Volumetric Analysis and Evaluation of Preoperative Metabolic Load

**DOI:** 10.3390/jcm15176693

**Published:** 2026-08-28

**Authors:** Oğuzhan Aydın, Yusuf Yunus Korkmaz, Murat Altay, İlyas Kudaş, Serap Baş, Özgür Bostancı, Erdem Kinaci

**Affiliations:** 1 Department of General Surgery, Basaksehir Cam and Sakura City Hospital, 34480 Istanbul, Turkey; 2 Department of Radiology, Basaksehir Cam and Sakura City Hospital, 34480 Istanbul, Turkey

**Keywords:** pancreaticoduodenectomy, new-onset diabetes mellitus (NODM), pancreatic volumetry, body mass index, remnant pancreas atrophy

## Abstract

**Objectives:** Advanced 3D volumetry is increasingly utilized to predict postoperative outcomes. It is widely hypothesized that remnant pancreatic volume and progressive atrophy dictate the loss of endocrine function after pancreaticoduodenectomy (PD). This study aims to investigate this anatomical paradigm by evaluating whether 3D volumetric tissue loss or the baseline metabolic load drives new-onset diabetes mellitus (NODM). **Materials and Methods:** This retrospective study included 159 non-diabetic patients who underwent PD. Preoperative and postoperative (6-month) computed tomography (CT) images were evaluated using Synapse 3D 6.7 version volumetric analysis software to calculate total pancreas volume, remnant volume, and postoperative progressive atrophy rate. All measured volumes were indexed to body weight. Factors associated with NODM were analyzed using multivariate logistic regression and ROC analysis. **Results:** Postoperative follow-ups revealed that 30.2% (*n* = 48) of the patients developed NODM. Interestingly, advanced 3D volumetric analysis revealed no significant differences between the diabetic and non-diabetic cohorts regarding body weight-indexed total pancreas volume, preoperative remnant volume, or postoperative progressive atrophy rate (*p* = 0.475). Instead, multivariate analysis identified the preoperative metabolic load, represented by Body Mass Index (BMI), as the sole independent predictor of NODM (*p* = 0.006, OR = 1.125), independent of surgical tissue loss. ROC analysis demonstrated a moderate predictive threshold for BMI > 26.5 kg/m^2^ (AUC = 0.644). **Conclusions:** In this cohort, the anatomical volume and progressive atrophy of the remnant pancreas, despite precise 3D quantification, were not statistically associated with the development of NODM after PD. Postoperative endocrine failure appears to be strongly associated with the patient’s baseline metabolic stress rather than solely the physical extent of surgical tissue loss. These findings suggest that while anatomical tissue preservation remains biologically important, preoperative metabolic risk stratification should be a clinical priority.

## 1. Introduction

Pancreaticoduodenectomy (PD) remains the primary surgical treatment method for periampullary region tumors. Although postoperative mortality rates have decreased below 5% due to advancements in surgical techniques and improvements in perioperative care, long-term morbidity factors have become critical elements determining patients’ quality of life [1,2]. Among these morbidity factors, one of the most remarkable is new-onset diabetes mellitus (NODM), also known as Type 3c diabetes, which develops as a result of the loss of the pancreas’s endocrine functions [3].

The incidence of NODM after PD is reported in the literature to be between 10% and 50% [3,4]. The etiology of this condition is based on mechanisms such as the loss of beta cell mass along with the resected pancreatic tissue, a decrease in pancreatic polypeptide secretion, and alterations in gastrointestinal hormones [2,5]. Traditionally, patient age, BMI, preoperative glycemic status, and underlying histopathology are accepted as the primary risk factors for the development of diabetes [2,4]. Current studies demonstrate that not only the amount of tissue removed during surgery but also the volume of the “remnant” pancreatic tissue plays a determining role in preserving endocrine function [1,6]. It is known that chronic obstruction developing after pancreaticoenteric anastomosis, the loss of trophic hormones, and vascular nutritional changes lead to progressive atrophy in the remnant tissue over time [5,7]. However, not only the volume of the remnant pancreas at the time of operation but also the progressive atrophy process it undergoes in the postoperative period affects functional outcomes [7]. Yet, the relationship between this dynamic atrophy process and the development of diabetes remains to be fully elucidated, especially in large patient series. On the other hand, with the development of CT-based pancreatic volumetry techniques in recent years, research has focused on “volumetric changes” beyond tissue loss [5,6].

Based on the “atrophy rate” calculated from preoperative and postoperative volumetric data, our study examines the relationship of this process with the development of diabetes independently of other clinical variables. As is known, the endocrine capacity of the pancreas is directly related not only to the absolute tissue volume but also to the metabolic load of the patient. Unlike many studies in the literature, our research takes into account the metabolic requirements of patients with different body masses; and aims to reveal the independent effect of body weight-indexed remnant pancreas volumes and their atrophy on the development of NODM.

## 2. Materials and Methods

### 2.1. Study Design and Patient Selection

**Figure 1:**
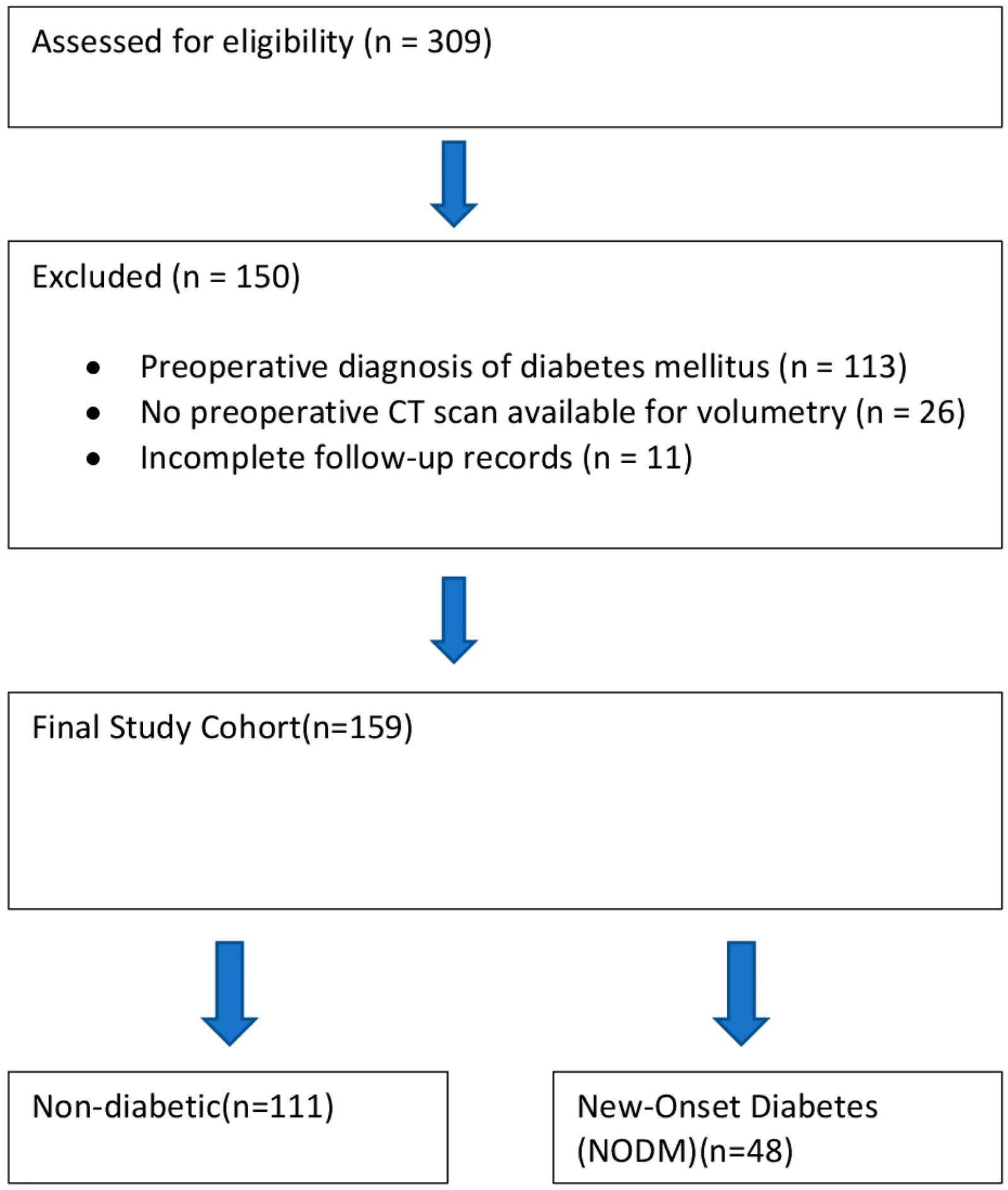
Study flow diagram of the patient screening and inclusion process.

This study was conducted by retrospectively analyzing the data of patients who underwent PD and had no preoperative diagnosis of diabetes between September 2020 and July 2025 at Basaksehir Cam and Sakura City Hospital. From an initial institutional cohort of 309 patients who underwent PD, 150 patients were excluded based on predefined criteria. Specifically, 113 patients were excluded due to a preexisting preoperative diagnosis of diabetes mellitus, 26 patients lacked accessible preoperative CT scans suitable for volumetric analysis, and 11 patients had incomplete follow-up records. Ultimately, a final cohort of 159 patients was included in the study and analyzed (Figure 1). Patients were divided into two main groups, “Diabetic” and “Non-diabetic,” based on their glucose metabolism status during the postoperative follow-up period.

### 2.2. Data Collection and Clinical Parameters

The patients’ demographic characteristics (age, gender), BMI, American Society of Anesthesiologists (ASA) physical status scores, and lengths of hospital stay were obtained from the hospital registry system. The diagnosis of diabetes was established based on at least two consecutive abnormal measurements of fasting blood glucose levels (≥126 mg/dL) or HbA1c levels (≥6.5%), or the clinical requirement for the initiation of glucose-lowering medication. To ensure a comprehensive assessment, glycemic status was monitored longitudinally up to 12 months postoperatively. NODM was categorized as early-onset (occurring within the first 3 months) or late-onset (occurring between 3 and 12 months). The 12-month postoperative glycemic follow-up was conducted as part of routine standard-of-care clinical practice. The final follow-up data for the most recently included patients were retrospectively extracted from the hospital registry upon the completion of their first postoperative year. Patients who developed early-onset NODM were included in the cohort, and their standardized 6-month postoperative CT scans were evaluated retrospectively to assess the ultimate stabilized anatomical state of the remnant pancreas. All 4 patients who died in the postoperative period (Clavien–Dindo Grade 5) within the study cohort were included in the analyses as they survived beyond the initial 30 days and their early glycemic follow-up data were available.

### 2.3. Radiological Evaluation and Volume Measurements

**Figure 2:**
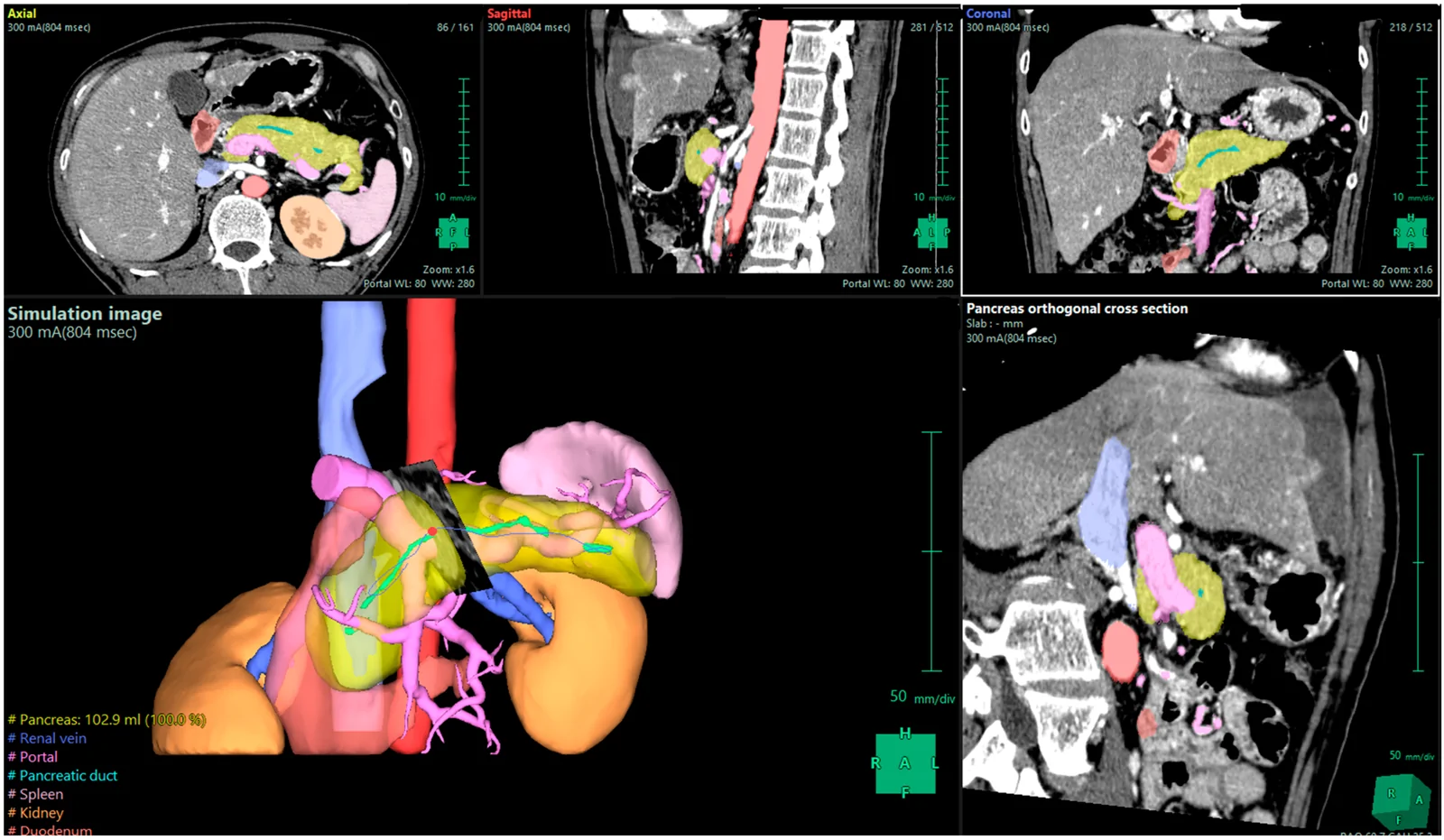
Three-Dimensional Calculation of Pancreatic Volume. A volumetric reconstruction generated by importing preoperative computed tomography (CT) images into the Synapse 3D software (Fujifilm). The multi-panel view illustrates axial, sagittal, coronal, and orthogonal cross-sections, alongside a 3D simulation image. In the 3D rendering, specific anatomical structures are color-coded to denote tissue boundaries: pancreas (yellow), pancreatic duct (cyan), portal vein (purple), renal vein (dark blue), spleen (light pink), kidney (orange), and duodenum (peach). The green orientation cubes display uppercase letters indicating anatomical planes (A: Anterior, P: Posterior, R: Right, L: Left, H: Head, F: Foot). In the 3D simulation, the red dot on the black orthogonal plane denotes the center of the cross-section axis. The colored area represent the specific tissue boundaries and the total volume calculated in cm^3^.

Preoperative and postoperative radiological images of all patients were reviewed via the digital archive system. Pancreatic volume measurements were performed using SYNAPSE 3D 6.7 version (Fujifilm Medical Systems) volumetric analysis software (Figure 2). Total pancreas volume, preoperative remnant pancreas volume, and postoperative remnant pancreas volumes were calculated as part of the evaluation. To accurately capture the stabilized parenchymal volume and effectively calculate the progressive atrophy rate, postoperative CT images were evaluated at an average of 6 months post-surgery (typically during routine oncological follow-up scans). The preoperative remnant pancreas volume was obtained by calculating the distal pancreas volume aligned with the left border of the SMV. The pancreatic atrophy rate was objectively quantified using the following mathematical formula: Atrophy Rate (%) = [(Preoperative Remnant Volume − Postoperative Remnant Volume)/Preoperative Remnant Volume] × 100. Additionally, to standardize physical and metabolic differences among patients; the measured total pancreas volume, preoperative remnant pancreas volume, and postoperative remnant pancreas volume values were individually divided by the patients’ preoperative body weights to calculate ‘Total Pancreas Volume Index (mL/kg)’, ‘Preoperative Remnant Volume Index (mL/kg)’, and ‘Postoperative Remnant Volume Index (mL/kg)’, which were then included in the statistical analyses. To minimize measurement bias, all volumetric assessments were performed by investigators blinded to the patients’ postoperative glycemic outcomes. Measurements were standardized using the portal venous phase of the contrast-enhanced CT scans to ensure optimal visualization of the pancreatic parenchyma. During postoperative measurements, particular attention was given to distinguishing true parenchymal tissue from postoperative edema or peripancreatic fibrotic changes, utilizing tissue density (Hounsfield Units) tracking features of the software.

### 2.4. Statistical Analysis

Data analysis was performed using SPSS v25.0 software. Descriptive statistics were presented as mean ± standard deviation (SD) for continuous variables, and as frequency (*n*) and percentage (%) for categorical variables. The Independent Samples *t*-test was used to compare continuous variables between the two groups, and relationships between categorical variables were analyzed using the Pearson Chi-Square test. Binomial Logistic Regression analysis was performed to identify independent risk factors predicting the presence of diabetes. ROC curve analysis was applied to determine the diagnostic accuracy and optimal cut-off points of the parameters in predicting diabetes. The statistical significance level was set at *p* < 0.05. To minimize selection bias and avoid model overfitting, a rigorous variable selection strategy was implemented for the multivariate analysis. Initially, all clinically relevant variables were assessed in the univariate analysis. Variables demonstrating a *p*-value of <0.05 in the univariate analysis were considered candidates for the multivariate logistic regression model. A backward stepwise elimination method was then employed. Given that the study observed 48 outcome events (NODM cases), we adhered to the standard ‘rule of ten’ events per predictor variable to prevent model overfitting. Consequently, our multivariate logistic regression model was carefully restricted to four candidate variables (BMI, age, atrophy rate, and underlying pathology) to ensure statistical stability.

### 2.5. Ethical Approval

Ethical approval for this study was obtained from the Clinical Research Ethics Committee of Basaksehir Cam and Sakura City Hospital with decision number [2025-263] dated [6 August 2025]. All procedures performed during the study were conducted in full compliance with the ethical standards of the 1964 Helsinki Declaration and its later amendments, as well as good clinical practice guidelines. Due to the retrospective design of the study, all data were obtained anonymously from the hospital registry system, ensuring maximum patient confidentiality.

## 3. Results

### 3.1. Demographic and Clinical Characteristics of the Patients

The mean age of the 159 patients included in the study was 59.36 ± 12.09 years (range: 20–85), with 63.5% (*n* = 101) males and 36.5% (*n* = 58) females. The patients’ mean BMI was calculated as 25.80 ± 4.45 kg/m^2^. Upon reviewing comorbidity statuses; Diabetes Mellitus (DM) developed in 30.2% (*n* = 48) of the patients. According to the ASA physical status classification; 8.2% (*n* = 13) of the patients were ASA 1, 57.2% (*n* = 91) ASA 2, 30.8% (*n* = 49) ASA 3, and 3.8% (*n* = 6) ASA 4. Evaluation of the histopathological data obtained after surgical resection of the 159 patients; revealed that the vast majority of cases were malignant tumors at 88.1% (*n* = 140), while 11.9% (*n* = 19) had benign pathologies.

Regarding volumetric measurements and clinical course parameters, the mean total pancreas volume was 96.10 ± 32.28 cm^3^ (range 30.8–226.7 cm^3^). The preoperative remnant pancreas volume was 47.1 ± 19.42 cm^3^ (range 8.4–130.4), while the postoperative remnant pancreas volume was determined as 34.14 ± 14.95 cm^3^ (range 5.2–84.4). The patients’ mean length of hospital stay was recorded as 19.76 ± 15.82 days. All descriptive data are presented in Table 1.

### 3.2. Comparison of Groups with and Without Diabetes

In the comparative analysis of demographic and clinical parameters between the diabetic and non-diabetic patient groups, a statistically significant difference was found regarding BMI values (*p* = 0.003). The mean BMI of diabetic patients was 27.36 ± 4.39 kg/m^2^, whereas the mean for non-diabetic patients was 25.13 ± 4.33 kg/m^2^.

No significant differences were detected between the groups in terms of age (*p* = 0.083), length of hospital stay (*p* = 0.532), and atrophy rate (*p* = 0.385). The underlying histopathological diagnosis (malignant or benign) did not have a statistically significant impact on the development of postoperative new-onset diabetes (*p* = 0.228). When volumetric measurements were examined, total pancreas volume (*p* = 0.685), preoperative remnant volume (*p* = 0.933), and postoperative remnant volume (*p* = 0.393) showed similar distributions in both groups. Furthermore, no significant relationship was found between the groups in terms of gender distribution (*p* = 0.593). When volumetric measurements were evaluated to assess their solitary impact on NODM, unadjusted logistic regression revealed no significant association for total pancreas volume (OR: 1.002, 95% CI: 0.992–1.013, *p* = 0.683) or preoperative remnant volume (OR: 0.999, 95% CI: 0.982–1.017, *p* = 0.933). Similarly, regarding the effects of radiologically calculated volumetric parameters indexed to patient body weight on diabetes development; no statistically significant differences were observed between the groups in terms of preoperative total pancreas volume index (*p* = 0.538), preoperative remnant volume index (*p* = 0.538), and postoperative remnant volume index (*p* = 0.129). The pancreatic atrophy rate observed during the follow-up period was also found not to be an independent risk factor for predicting diabetes development (*p* = 0.475). Conversely, higher preoperative BMI values demonstrated a strong statistical relationship with postoperative diabetes development (*p* < 0.05). Our findings are summarized in Table 2.

### 3.3. Multivariate Analysis of Diabetes Risk Factors

To identify independent factors associated with the presence of New-Onset Diabetes Mellitus (NODM) and to avoid mathematical coupling between variables, a multivariable binary logistic regression model was constructed using absolute (non-indexed) volume measurements. To prevent multicollinearity among the highly correlated anatomical variables, absolute preoperative remnant volume was selected alongside atrophy rate, age, and BMI. According to the analysis results, BMI was determined to be the sole independent significant predictor for the presence of diabetes (*p* = 0.006). The model indicated that each one-unit increase in BMI raises the risk of diabetes by approximately 12.5% (OR = 1.125; 95% CI: 1.034–1.224). Absolute preoperative remnant volume (*p* = 0.522), pancreatic atrophy rate (*p* = 0.459), and patient age (*p* = 0.207) did not show an independent association with diabetes development in the multivariable model (Table 3).

### 3.4. ROC Analysis Results

In the study, ROC curve analysis was performed to determine the predictive capacities of clinical parameters for the presence of NODM. The analysis revealed that BMI possesses statistically significant discriminatory power in predicting NODM (AUC = 0.644). On the other hand, the pancreatic atrophy rate did not have significant discriminatory power in predicting NODM, with results bordering on chance (AUC = 0.536).

In the detailed coordinate analysis for BMI, the cut-off points providing the optimal balance in predicting NODM risk, along with their sensitivity and specificity values, are presented in Table 4.

## 4. Discussion

The loss of endocrine functions following PD and the subsequent development of NODM is one of the most critical long-term morbidity elements that determine postoperative quality of life. The incidence of NODM after PD is reported in the literature to be between 10% and 35% [3,8,9,10,11]. In our study, postoperative diabetes developed in 30.2% (*n* = 48) of the 159 patients without preoperative diabetes, which is consistent with current literature data.

The most striking finding of this study is the demonstration that new-onset diabetes developing after PD is directly related to the patient’s preoperative metabolic baseline rather than the absolute or weight-indexed volume of the remnant pancreatic tissue [12]. Although increasingly common volumetric studies in the literature propose remnant volume and progressive atrophy as the primary causes of endocrine insufficiency [13,14]; our results reveal that anatomical deficiency or atrophy rate alone is not the determining factor. No significant relationship was found between body weight-indexed total and remnant pancreas volumes and the development of NODM. Attributing the relationship between an elevated BMI and NODM solely to the paradigm of peripheral insulin resistance combined with surgical beta-cell loss may be an oversimplification. The underlying pathophysiology is highly complex and multifactorial. Preoperative obesity contributes to chronic low-grade systemic inflammation, lipotoxicity, and altered adipokine secretion, all of which progressively impair residual beta-cell function. Furthermore, the gastrointestinal reconstruction inherent to PD fundamentally alters the entero-insular axis. The bypass of the duodenum significantly modifies the incretin effect (such as GLP-1 and GIP secretion). When this compromised incretin response is superimposed on a state of obesity-induced metabolic stress and an abruptly reduced active endocrine mass, the remaining beta-cells fail to mount an adequate compensatory response, thereby precipitating clinical diabetes [15]. This indicates that in the presence of obesity and metabolic syndrome, patients should be considered at high risk for NODM, regardless of how well the remnant pancreas volume is preserved or how low the atrophy rate is. However, it is crucial to note that the lack of a statistically significant association in our cohort does not imply that pancreatic tissue loss is biologically irrelevant. The physical reduction of beta-cell mass undoubtedly plays a fundamental role in endocrine failure; yet, in our cohort, this anatomical effect may have been statistically overshadowed by the profound impact of preexisting metabolic stress (BMI).

Another significant finding of our study is that the underlying histopathological diagnosis was not found to have an independent effect on the development of NODM. Literature suggests that pancreatic adenocarcinoma is associated with glucose intolerance and diabetes through paraneoplastic factors secreted by the cells [16,17]. However, our findings show that the diabetic course in the postoperative period after tumor resection is driven by the patient’s basal metabolic state (BMI) rather than specific tumor biology [12]. This highlights that all PD patients with a high preoperative BMI, regardless of malignancy or benign pathology, should be considered equally high risk for postoperative endocrine insufficiency and followed closely.

According to our results, multivariate analysis identified BMI as the only independent risk factor predicting the NODM. Our analysis shows that each one-unit increase in BMI raises the risk of NODM by approximately 11.9%. This can be interpreted as obesity and associated insulin resistance placing an additional burden on the limited insulin secretion capacity post-surgery, thereby accelerating the deterioration of glycemic balance. Similar studies in the literature have also noted that preoperative metabolic status, rather than anatomical volume, may be the determinant in the development of NODM [2,15].

Our ROC curve analysis demonstrated that a BMI value of >26.5 kg/m^2^ is an optimal cut-off point for identifying patients at higher risk of NODM. However, it is important to acknowledge that its discriminatory capacity remains moderate (AUC = 0.644). With both sensitivity and specificity at approximately 60%, this threshold should not serve as a definitive, standalone diagnostic tool. Instead, it represents a practical clinical alert, signifying a heightened metabolic risk profile that requires surgeons to adopt a more vigilant approach in postoperative glycemic monitoring. Conversely, it was determined that the atrophy rate did not have significant discriminatory power in predicting the presence of diabetes, showed no statistical significance. hese findings suggest that BMI, a simple clinical indicator, may serve as a more practical clinical adjunct compared to complex volumetric analyses for estimating NODM risk in patients planned for Whipple surgery.

Our study has several limitations that should be acknowledged. First, its retrospective design may limit the generalizability of the results. Second, the relatively small number of outcome events (*n* = 48) precluded a statistically robust sub-stratification and comparative analysis of early-onset (0–3 months) versus late-onset (3–12 months) NODM. Consequently, our findings represent the overall cumulative risk during the first postoperative year, and we could not systematically sub-analyze transient perioperative stress hyperglycemia against persistent diabetes in early-onset cases. Third, and perhaps most importantly, the lack of routine preoperative screening tools—such as HbA1c, fasting insulin levels (HOMA-IR), or a detailed family history of diabetes—means that unmeasured metabolic confounding is possible. Because we could not systematically rule out preexisting dysglycemia for every patient, some patients in the cohort might have had undiagnosed subclinical prediabetes; therefore, BMI should be interpreted as a surrogate risk marker for underlying metabolic stress rather than a strictly proven causal factor. Fourth, the statistical use of a stepwise regression model carries inherent limitations regarding variable selection bias, although we mitigated this by adhering to the event-per-variable rule. Fifth, the 3D volumetric measurements were performed by a single investigator; the absence of formal interobserver and intraobserver reproducibility analyses is a methodological limitation. Finally, while a BMI of >26.5 kg/m^2^ was identified as an optimal cut-off, its clinical discriminatory value remains moderate (AUC = 0.644). Therefore, rather than serving as a standalone validated diagnostic tool, this threshold should be utilized as a practical clinical alert to identify patients requiring more vigilant postoperative glycemic monitoring.

In conclusion, our study demonstrated that preoperative BMI is the strongest predictor of NODM after PD. There was no direct relationship found between preoperative pancreas volume, remnant volume or the rate of postoperative atrophy and NODM. In light of these findings, identifying and managing preoperative metabolic risks should be considered a critical component of patient care, serving as an important complement to anatomical tissue preservation efforts.

## Figures and Tables

**Table 1:** Baseline demographic and clinical characteristics of the study cohort (*n* = 159).

Variable	Frequency (*n*)/Mean	Percentage (%)/SD (±)	Range (Min–Max)
Age (years)	59.36	±12.09	20–85
Gender (Male/Female)	101/58	63.5%/36.5%	-
BMI (kg/m^2^)	25.80	±4.45	12.41–43.36
ASA Score (1/2/3/4)	13/91/49/6	8.2%/57.2%/30.8%/3.8%	-
Pathology (Benign/Malign)	19/140	11.9%/88.1%	-
Operation Type (PD/PPPD)	118/41	74.2%/25.8%	-
Preoperative Drainage (No/PTBD/ERCP)	66/87/6	41.5%/54.7%/3.8%	-
Pancreatic Fistula (No-Biochemical leak/B/C)	117/41/1	73.6%/25.8%/0.6%	-
Hospital Stay (days)	19.76	±15.82	7–138
Total Pancreas Volume (cm^3^)	96.10	±32.28	30.80–226.70
Preoperative Remnant Volume (cm^3^)	47.01	±19.42	8.4–130.4
Postoperative Remnant Volume (cm^3^)	34.15	±14.95	5.2–84.4
Atrophy Rate (%)	26.30	±21.03	0–84.75

**Table 2:** Comparison of clinical and radiological parameters between diabetic and non-diabetic patients.

Variable	DM (−) (*n* = 111)	DM (+) (*n* = 48)	*p*-Value
Age (years)	58.27 ± 12.79	61.90 ± 9.96	0.083
Gender (Male/Female), *n*	72/39	29/19	0.593
BMI (kg/m^2^)	25.13 ± 4.33	27.36 ± 4.39	0.003 *
Operation Type (PD/PPPD), *n*	81/30	37/11	0.587
Pancreatic Fistula (No/Yes), *n*	85/26	32/16	0.171
Complications (Clavien–Dindo ≥ 3), *n*	25	12	0.734
Hospital Stay (days)	19.24 ± 16.27	20.96 ± 14.83	0.532
Total Pancreas Volume (cm^3^)	95.42 ± 32.23	97.69 ± 32.71	0.685
Preoperative Remnant Volume (cm^3^)	47.18 ± 19.71	46.90 ± 18.96	0.933
Postoperative Remnant Volume (cm^3^)	34.81 ± 15.02	32.60 ± 14.83	0.393
Atrophy Rate (%)	25.35 ± 20.38	28.51 ± 22.52	0.385
Total Pancreas Volume Index (mL/kg)	1.36 ± 0.41	1.32 ± 0.46	0.538
Preop Remnant Volume Index (mL/kg)	0.67 ± 0.26	0.63 ± 0.26	0.538
Postop Remnant Volume Index (mL/kg)	0.51 ± 0.21	0.44 ± 0.19	0.129
Pathology (Benign/Malign), *n*	11/100	8/40	0.228

*: There was statistically significant data.

**Table 3:** Multivariate logistic regression analysis of predictors for diabetes mellitus.

Variable	B	*p*-Value	Odds Ratio (OR)	95% CI for OR
BMI (kg/m^2^)	0.118	0.006 *	1.125	1.034–1.224
Age	0.021	0.207	1.021	0.988–1.055
Atrophy Rate (%)	0.007	0.459	1.007	0.989–1.055
Preoperative Remnant Volume (cm^3^)	−0.007	0.522	0.993	0.974–1.014

*: There was statistically significant data.

**Table 4:** ROC curve analysis results for predicting NODM mellitus.

Variable	AUC	95% CI	*p*-Value	Cut-Off	Sensitivity (%)	Specificity (%)
BMI (kg/m^2^)	0.644	0.551–0.737	0.004 *	>26.5	60.4	61.3
Atrophy Rate (%)	0.536	0.434–0.638	0.472	-	-	-

*: There was statistically significant data.

## Data Availability

The data presented in this study are available on request from the corresponding author.
